# The Drosophila phenotype ontology

**DOI:** 10.1186/2041-1480-4-30

**Published:** 2013-10-18

**Authors:** David Osumi-Sutherland, Steven J Marygold, Gillian H Millburn, Peter A McQuilton, Laura Ponting, Raymund Stefancsik, Kathleen Falls, Nicholas H Brown, Georgios V Gkoutos

**Affiliations:** 1 FlyBase, Department of Genetics, University of Cambridge, Downing Street, Cambridge, UK; 2 Department of Genetics, University of Cambridge, Downing Street, Cambridge, UK; 3 Gurdon Institute & Department of Physiology, Development and Neuroscience, University of Cambridge, Tennis Court Road, Cambridge, UK; 4 The Biological Laboratories, Harvard University, 16 Divinity Avenue, Cambridge, MA, USA

**Keywords:** Drosophila, Phenotype, Ontology, OWL, OBO, Gene ontology, FlyBase

## Abstract

**Background:**

Phenotype ontologies are queryable classifications of phenotypes. They provide a widely-used means for annotating phenotypes in a form that is human-readable, programatically accessible and that can be used to group annotations in biologically meaningful ways. Accurate manual annotation requires clear textual definitions for terms. Accurate grouping and fruitful programatic usage require high-quality formal definitions that can be used to automate classification. The *Drosophila* phenotype ontology (DPO) has been used to annotate over 159,000 phenotypes in FlyBase to date, but until recently lacked textual or formal definitions.

**Results:**

We have composed textual definitions for all DPO terms and formal definitions for 77% of them. Formal definitions reference terms from a range of widely-used ontologies including the Phenotype and Trait Ontology (PATO), the Gene Ontology (GO) and the Cell Ontology (CL). We also describe a generally applicable system, devised for the DPO, for recording and reasoning about the timing of death in populations. As a result of the new formalisations, 85% of classifications in the DPO are now inferred rather than asserted, with much of this classification leveraging the structure of the GO. This work has significantly improved the accuracy and completeness of classification and made further development of the DPO more sustainable.

**Conclusions:**

The DPO provides a set of well-defined terms for annotating *Drosophila* phenotypes and for grouping and querying the resulting annotation sets in biologically meaningful ways. Such queries have already resulted in successful function predictions from phenotype annotation. Moreover, such formalisations make extended queries possible, including cross-species queries via the external ontologies used in formal definitions. The DPO is openly available under an open source license in both OBO and OWL formats. There is good potential for it to be used more broadly by the *Drosophila* community, which may ultimately result in its extension to cover a broader range of phenotypes.

## Background

### Drosophila Phenotype Ontology (DPO)

*Drosophila melanogaster* is one of the most widely used model organisms for genetics, with a wealth of genetic and phenotypic data generated over the past hundred years. FlyBase, the model organism database for *Drosophila* genetics, curates and maintains a near-comprehensive set of records of non-molecular *Drosophila* phenotypes using a combination of formal annotation strategies and free text. Formal curation of phenotypes takes one of two forms: phenotypes affecting specific anatomical structures are curated using terms from the *Drosophila* anatomy ontology (DAO) [1]; other phenotypes, including those affecting behaviour and biological processes such as cell division, are curated using terms from the *Drosophila* Phenotype Ontology (DPO), which is limited to a relatively small number (<200) of high-level and commonly described phenotypic classes. To date, this ontology has been used to annotate over 159,000 phenotypes. It is openly available under a Creative Commons attribution license (CC-BY) in both OBO and OWL formats (See 1 for download options).

**Table 1:** Accessing the DPO

**Target**	**Base URL extension**
Homepage	fbcv
Term request tracker	fbcv/tracker
Pre-reasoned OBO version	fbcv/dpo-simple.obo
Full OWL version	fbcv/dpo-non-classified.owl
Full details of all available versions	fbcv/downloads
Individual term details for FBcv_0000423	FBcv_0000423

DPO files, content and related resources can all be accessed via Persistent URLs (PURLs). The base URL for all PURLS is http://purl.obolibrary.org/obo/. Column 1 describes the targets of various PURLs specified in Column 2 as extensions to this base URL. Individual term details resolve to html when viewed in a browser, but resolve to XML when accessed programatically.

### Biomedical ontologies

Biomedical ontologies are queryable classifications of biological entities such as anatomical structures, processes, behaviours and phenotypes. They are commonly used by bioinformatics resources to provide controlled vocabularies for annotating a range of entities (such as research papers, genes and genotypes) with assertions about, for example, gene function, phenotypes and gene expression patterns [2-5]. Class and part hierarchies in ontologies provide terms with a range of specificity allowing curators to choose an appropriately specific term depending on the information available. Term names on their own are frequently ambiguous, so textual definitions of terms are needed to ensure consistent and accurate manual annotation.

The semantics of ontologies are used to group annotations in biologically meaningful ways. Typically, this is done by grouping annotations using class and part hierarchies (partonomy). For example, a query for genes expressed in the *Drosophila* leg could return gene expression annotated with the term middle leg (a subclass of leg) and claw (a part of the leg) as well as with the term leg. The usefulness of such grouping depends on the accuracy of classification and of assertions about partonomy.

Most highly-used biomedical ontologies have been developed in OBO format [6]. Historically, these ontologies have been poorly formalised and manually maintained. Improvements to the expressiveness of OBO format and the definition of OBO format semantics via mapping to OWL2 [6,7] have made it possible to formalise definitions so that OWL reasoners can be used to automate classification, check for consistency and run queries. Where formal definitions reference terms from external ontologies, OWL reasoners can leverage the formal structure of ontologies from which terms are imported to automate classification, check consistency and run queries. This approach is already being used to improve the GO [8,9], the DAO and a number of phenotype ontologies [10-12]. Improved formalisation can also make more sophisticated systems for grouping annotations and querying ontology content possible. For example, the Virtual Fly Brain project (VFB) [13,14] uses a set of custom formalisations for representing neuroanatomy to drive custom queries and to enrich the results of queries of expression and phenotype data.

When coupled with modularisation [15], formalisation can facilitate integrative approaches to reason and compare across disparate species. For example, the PhenomeNet approach aligns phenotypes across species and enables the generation of a single, unified, and logically consistent representation of phenotype data for multiple species. By combining the anatomy and phenotype ontologies of six species (yeast, worm, fly, mouse, zebrafish, rat) alongside human disease phenotypes, PhenomeNet generates a cross-species network of phenotype similarity between genotypes and diseases [16].

### PATO

The Phenotype And Trait Ontology (PATO) [17] is an ontology of phenotype-related qualities that comprise the basic entities that we can perceive and/or measure such as color, size, mass, length etc. Qualities inhere in entities: every entity comes with certain qualities that exist as long as the entity exists. PATO allows for the description of affected entities by combining various ontologies that describe the entities that have been affected, such as the various anatomical ontologies, the GO [18] and the Cell Ontology [19], with the various qualities it provides for defining how these entities were affected. For instance, to describe a brown eye phenotype, we could combine the PATO term *brown* with an anatomy ontology term for an *eye*.

### Defining and formalising the DPO

Many of the terms that make up the DPO were originally developed and maintained in an informal hierarchy [20]. This became an explicit classification hierarchy following the adoption of OBO format *circa* 2006 but initially no further formalisation was added. No textual definitions were provided for terms in the original hierarchy, and this remained the case until recently. We have now developed textual definitions for all DPO terms and formal definitions in OWL for 77% of them. Here we describe the results of this work and how it has improved the accuracy of the ontology, its usefulness for grouping and querying annotations, and its potential utility in cross-species querying of phenotypes.

## Results

Defining terms that are already widely used is challenging. New definitions either need to be consistent with existing annotations or existing annotations need to be updated to conform to new definitions. To ensure consistency between the new DPO definitions and existing annotation, the process of developing definitions involved collaboration between ontology developers and curators, making use of both the tacit knowledge of curators and the extensive free-text descriptions of phenotypes in FlyBase. During this process, we discovered inconsistencies in existing annotations and invested considerable effort to correct these and, where necessary, to modify annotations to conform to new terms.

We have largely followed formalisation patterns developed for other phenotype ontologies [10-12,21] with all phenotypes being subclasses of PATO quality and particular qualities having an **inheres_in** (RO_0000052) relationship to some entity class. Types of entity are referred to using terms from other widely-used bio-ontologies such as the GO [18] and the cell ontology (CL) [19]. Re-using standard patterns provides interoperability with both the entity ontologies and other phenotype ontologies, providing good potential for more sophisticated queries of *Drosophila* data and for cross-species querying.

Broadly, a phenotype can be defined as an observable attribute of an organism. However, model organism geneticists, such as those working in *Drosophila* genetics, typically use the term phenotype to refer to an abnormality in some anatomical structure, process or behavior in a specified genotype compared to wild-type. Accordingly, we define phenotype, the root term of our ontology, as a *quality* (PATO_0000001) of some anatomical structure, process or behavior that differs from wild-type. Following the definition pattern developed for other phenotype ontologies (e.g. [21]), we record this formally using a 'qualifier’ relationship to the PATO term *abnormal* (PATO_0000460): 

phenotype *EquivalentTo* quality *that ***qualifier ***some* abnormal

We have not attempted to formalise the comparative nature of phenotypes more explicitly. All phenotypes in the DPO are manually classified under this root term and so inherit the assertion of abnormality.

### Processual and Behavioral abnormalities

The DPO contains a variety of terms that describe phenotypes that are defects in biological processes. For such terms, a GO process term is linked to a PATO term that describes how this process was affected.

For example, the DPO term *radiation resistant* (FBcv_0000439) is defined as a *decreased sensitivity of a process* (PATO_0001552), inhering in *response to radiation* (GO_0009314): 

'radiation resistant’ *EquivalentTo* 'decreased sensitivity of a process’ *that ***inheres_in ***some* 'response to radiation’

By using the GO to define processual abnormalities, we can leverage classification within it to infer much of the classification of processual phenotypic classes. This has led to new classifications not originally present in the original, asserted classification. For example, *stress response defective* (FBcv_0000408) originally had only 2 asserted subclasses. We now define it using the GO term *response to stress* (GO_0006950): 

'stress response defective’ *EquivalentTo* phenotype *that ***inheres_in ***some* 'response to stress’ *and ***qualifier ***some* abnormal

**Figure 1:**
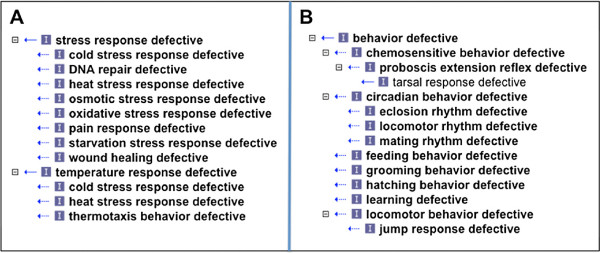
**Autoclassification of processual phenotypes.** Auto-classification of processual phenotypes, leveraging the GO. Terms in bold have *equivalent class* definitions. **Panel A** shows classification of stress response phenotypes. **Panel B** shows a portion of the behavioral phenotype classification.

After auto-classification, this class has 8 subclasses (see Figure 1A) including a number, such as *DNA repair defective* (FBcv_0000423), that were not initially obvious.

Other inferred subclasses of *stress response defective* have additional inferred superclasses. For example, *cold stress response defective* (FBcv_0000684) is an inferred subclass of both *stress response defective* (FBcv_0000408) and *temperature response defective* (FBcv_0000683). Maintaining such multiple classification by hand is well known to be difficult, error prone and poorly scalable. Auto-classification based on assertion of properties is much less error prone and can scale well [22].

The DPO also contains a range of terms for behavioral phenotypes (Figure 1B We define a grouping class *behavior defective* (FBcv_0000387) using the GO term *behavior* (GO_0007610) 

'behavior defective’ *EquivalentTo* quality *that ***inheres_in ***some* behavior *and ***qualifier ***some* abnormal

This allows us to defer the thorny decision of what to class as behavior [23] to the GO. With automated classification, this has resulted in a number of classes being moved out from under the behavioral classification. This includes a set of terms that refer to defects in perception, which the GO classifies as a neurological process but not as behavior. It also includes the general class *'circadian rhythm defective’* (FBcv_0000394), originally classified under behavior defective because circadian rhythm defects are commonly assayed using behavior. However, many non-behavioral processes are under circadian control. We have added a new term, *circadian behavior defective * (FBcv_0000679) for specifically behavioral circadian phenotypes.

For processual and behavioral phenotypes, the evidence for disruption is commonly indirect. A defect in the process of segmentation during embryogenesis might be inferred from disruption to segmental pattern in the cuticle, formed many hours after the segmentation process, with many developmental processes acting in between. Likewise, the disruption of a behavioral reflex might be inferred from the absence of a reflex reaction, but this absence could also be due to disruption of muscles or sensory perception. With appropriate extra evidence and controls, the case for disruption of the process or behavior can be compelling, but in the absence of this, it may be more appropriate to simply record the directly observed phenotype. A system for recording phenotypes from the literature has to cater for both types of assertion. Where the evidence is an observation of anatomy, this can be recorded directly using the the *Drosophila* anatomy ontology. Where the evidence is an observation of an animal’s movement, we give annotators a choice of DPO terms, one of which is neutral about whether the phenotype is behavioral. For example, the *jump response* (GO_0007630), a well characterised reflex escape response behavior in flies, is used to define the term *jump response defective*. Annotation to *jump response defective* is only warranted if flies fail to jump in a standard assay for this reponse, and controls have been done which discount simple physical explanations, such as defective legs or leg musculature. A broader term, *jumping defective* (FBcv_0000415), is available for cases where no such controls are in place.

#### Automated textual definitions for processual and behavioral phenotypes

To keep definitions up-to-date with changes in the GO, we automatically derive human-readable textual definitions from GO terms for classes defined using the pattern: 

*EquivalentTo* quality *that ***inheres_in ***some* <GO process class>*and ***qualifier ***some* abnormal 

For example, *stress response defective* gets the textual definition: 

“A phenotype that is a defect in 'response to stress’ (GO_0006950). The GO term 'response to stress’ is defined as: 'Any process that results in a change in state or activity of a cell or an organism (in terms of movement, secretion, enzyme production, gene expression, etc.) as a result of a disturbance in organismal or cellular homeostasis, usually, but not necessarily, exogenous (e.g. temperature, humidity, ionizing radiation).’”

We only use this mechanism for terms that do not have a manually supplied definition. We do not use it where formal definitions use more specific PATO terms, as it has proven difficult to reliably derive human readable definitions for these cases.

### Phenotypes of cells and multi-cellular structures

The DPO contains a number of terms for cell phenotypes such as *increased cell size* (FBcv_0000363), *increased cell number* (FBcv_0000362) and *cell death defective* (FBcv_0000425). We define these with reference to the cell type ontology term *cell* (CL_0000000) or to some subclass of *cellular process* (GO_0009987).

An *increased cell size* phenotype can be the result of a variety of abnormal underlying biological processes including defects in cell growth or changes in the rate of cell division. In the absence of evidence for an underlying mechanism, curators need to be able to record this observation directly. We therefore define *increased cell size* using the terms *increased size* (PATO_0000586) and *cell* (CL_0000000): 

'increased cell size’ *EquivalentTo* 'increased size’ *that ***inheres_in ***some* cell

The DPO class *increased cell number* was originally classified as a subclass of *size defective* (FBcv_0000357). But there is a complicated relationship between size and cell number: an increase in cell number need not result in larger size if it is accompanied by a decrease in cell size. An increase in cell number is a phenotype that can only be exhibited by a *multicellular structure* (FBbt_00100313). We define it using *has extra parts of type * (PATO_0002002)^a^ and cell (CL_0000000) as follows: 

'increased cell number’ *EquivalentTo* 'has extra parts of type’ **towards ***some* cell and **inheres_in ***some* 'multicellular structure’

The phenotype *cell death defective * (FBcv_0000425) provides an interesting example of the difficulty of defining widely-used terms based on their names alone. We initially defined this class using *programmed cell death* (GO_0012501) as: 

*EquivalentTo* quality *that ***inheres_in ***some* 'programmed cell death’ *and ***qualifier ***some* abnormal

But analysis of free text phenotype descriptions and feedback from curators quickly made it clear that existing usage consisted of cases where the amount of cell death occurring in one or more multicellular structures was abnormal. In many cases it was not clear whether this was due to a defect in regulation of cell death in the tissue or due to a defect in the core processes of cell death. So, we instead chose to define this class as a union: 

*EquivalentTo* (quality *that ***inheres_in ***some* 'programmed cell death’ *and ***qualifier ***some* abnormal) OR (quality *that ***inheres_in ***some* 'regulation of programmed cell death’ *and ***qualifier ***some* abnormal)

### Lethality and stage

Following typical usage by *Drosophila* geneticists, we use the term *lethal * (FBcv_0000351) to refer to a phenotype in which, to a good approximation, all animals in a population do not survive to become mature adults. We use *'partially lethal - majority die’* (FBcv_0000352) (AKA semi-lethal) to refer to a phenotype where most but not all animals die before mature adulthood: 

*lethal*: “A phenotype of a population that is the death of all animals in that population at some stage or stages prior to becoming a mature adult.”

*partially lethal - majority die*: “A phenotype of a population that is the death a majority of animals in that population prior to becoming a mature adult.”

To record that animals die before mature adulthood says nothing about the stages of development when death occurs, but this information is of great practical importance. Geneticists working on stages before mature adulthood need to be able to find genotypes that survive to a stage suitable for their experiments. Knowing the various stages at which significant number of animals of a particular genotype die can also be useful in allowing researchers to home-in on stages to characterise for defects.

FlyBase historically recorded information about the stages of death due to specific genotypes in a semi-controlled form by combining terms like 'lethal’ and 'semi-lethal’ with terms from an ontology of developmental stage. However, the semantics of these combinations were never codified and so the resulting annotations in FlyBase were not reliably useful for queries about the stages at which death occurs.

We devised a set of phenotype terms, with a formal semantics in OWL, for recording and reasoning about the stages at which death occurs in a population. Our aim was a system that separated assertions about the percentage of animals dying at various stages, which could apply to populations with significant adult survivors, from use of the term 'lethal’, which always refers to the lack of survival to mature adulthood.

These phenotype terms are defined using a combination of four elements. First we define a population of *Drosophila* using a term from the DAO, *organism* (FBbt_00000001) for an individual member of the species. 

'population of Drosophila’ *EquivalentTo *

population

*that* (**has_member***some* organism)

*and* (**has_member***only* organism)

**Figure 2:**
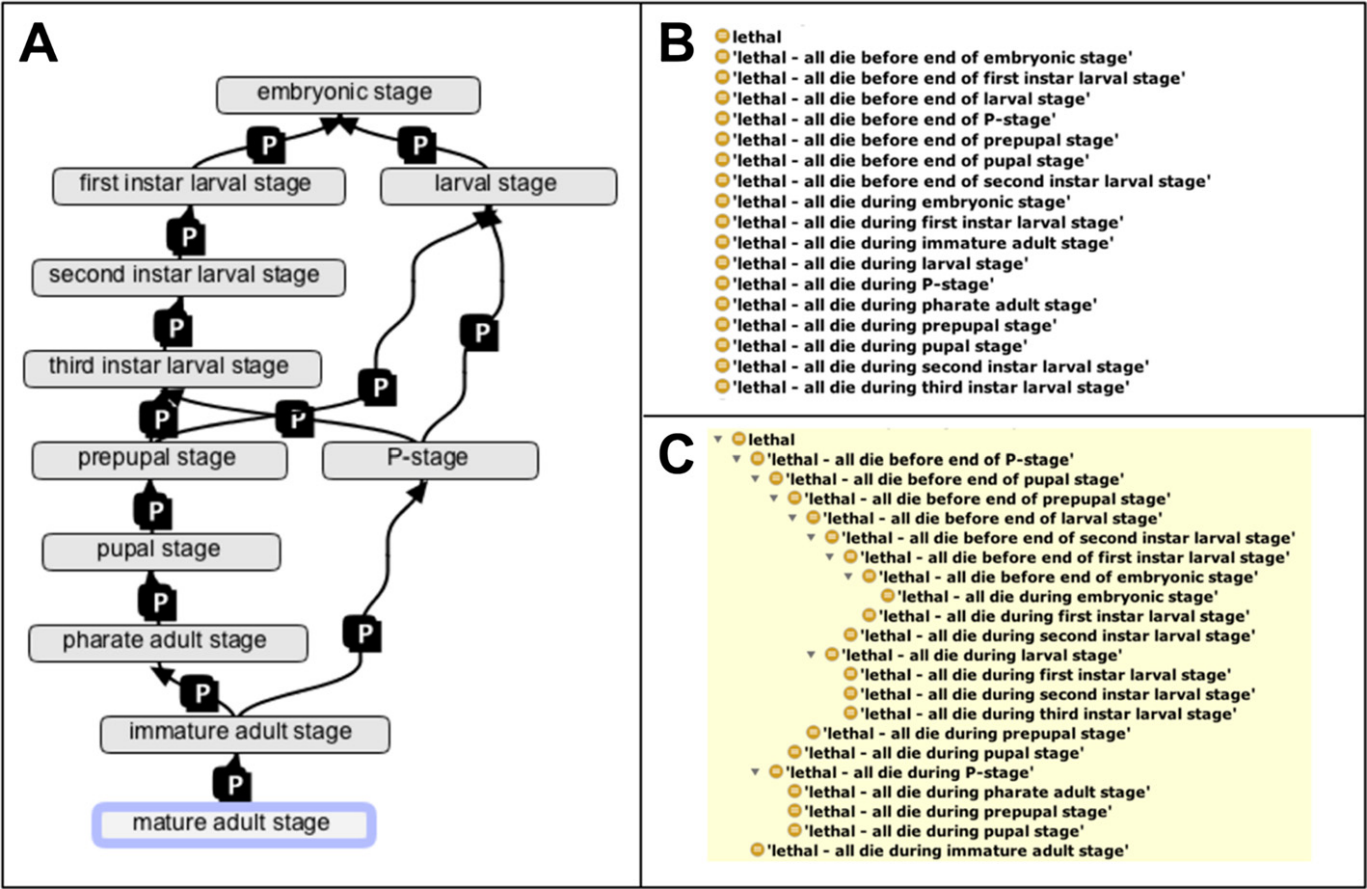
**Lethal phase phenotypes.** When do they die? - classes for recording and reasoning about the timing of death in lethal phenotypes. - **Panel A** shows the temporal relationships between *Drosophila* life stages from the *Drosophila* stage ontology. The P icon stands for **immediately_preceded_by**, which corresponds to the Allen relation 'meets’. **Panel B** shows a set of lethal phenotype terms prior to auto-classification. **Panel C** shows the same set of classes after auto-classification using the HermiT reasoner.

To define the stage or age of members of a population, we use a set of terms for life stages from the *Drosophila* stage ontology (http://purl.obolibrary.org/obo/fbdv.owl) (see Figure 2A) along with a set of relations and axioms for reasoning about relative timing based on a subset of the Allen Interval Algebra [24]. For example, we can refer to a population of *Drosophila* in which all members are younger than the third instar larval stage using: 

'population of Drosophila’

*that ***has_member ***some* (organism *and* ((**has_age ***some* (**precedes ***some* 'third instar larval stage’))))

*and* (**has_member ***only* (organism *and* (**has_age ***some* (**precedes***some* 'third instar larval stage’))))^b^

Finally, we use an OWL data property, **has_increased_mortality_rate**, to record the overall percent mortality rate, excluding the wild-type death rate for the stage in question^c^. An alternative approach would be to use a data property designed to record the penetrance of any phenotype. But there is, to our knowledge, no logically sound way to link percent penetrance recorded using an OWL data property to a specific phenotype. In contrast, using **has_increased_mortality** allows us to specify a phenotype and its penetrance in a single assertion, so linking the two is not an issue. Expressing death rates as an increase over wild-type allows this system to be used to define the phenotypic class *short lived* (FBcv_0000385) - which needs to cover stages of life for which wild-type death rates will be high.

The root term *increased mortality* (FBcv_0002004) is defined, without any stage restriction, as: 

“A phenotype that is an increase in the rate of death in a population at any any stage of life (during development or adulthood), over the rate seen in a wild-type control population.”

It has a formal definition that specifies a minimum increase in mortality of 5%: 

'increased mortality’ *EquivalentTo* (

**has_increased_mortality_rate***some* short [>=5]

*and ***inheres_in ***some* 'population of Drosophila’)

'increased mortality’ *SubClassOf* phenotype

For this modelling approach to work, we need a hard, non-zero cut-off and chose 5% as a reasonable figure based on community usage. These hard percentage cut-offs are considered rules-of-thumb for annotators, rather than strict rules as sometimes only qualitative assertions are available. Similarly, we use a cutoff of 98% for defining 'lethal’ to take into account that this term is used even when there are rare 'escaper’ animals that survive to adulthood: 

lethal *EquivalentTo* (

**has_increased_mortality_rate***some* short[<= 98]) *that ***inheres_in ***some* (

'population of Drosophila’

*that ***has_member ***some* (organism *that ***has_age** some (**precedes ***some* 'mature adult stage’))

*and ***has_member ***only* (organism *that ***has_age ***some* (**precedes** some 'mature adult stage’)))

For each of the major life stages, we define a term for recording that some animals (>= 5%) die during that stage and another for recording that some animals die before the end of that stage. We define similar pairs of terms for recording that most (>= 50%) or that 'all’ (>= 98%) die during or before the end of a specified stage.

We also define terms for partial lethality: 

partially lethal - majority die *EquivalentTo* (**has_increased_mortality_rate ***some* short[>50, <= 98]) *that ***inheres_in ***some* ('population of Drosophila’ *that ***has_member ***some* (organism *that ***has_age** some (**precedes ***some* 'mature adult stage’)) and **has_member ***only* (organism *that ***has_age ***some* (**precedes** some 'mature adult stage’)))

The resulting list of terms is completely flat prior to reasoning, but forms a deeply nested classification following OWL reasoning. Figure 2 shows subclasses of the term 'lethal’ before and after reasoning, illustrating a small part of this inferred classification. Simply grouping annotations using the class hierarchy generated by classification with an OWL reasoner, one can query for genotypes that cause all (>=98%) animals to die before some specified stage, or significant numbers of animals to die before or during some specified stage. This allows, for the first time, accurate grouping of DPO annotations based on the stage of lethality.

We are in the process of converting our existing annotation set of over 30,000 lethal and semi-lethal phenotype assertions to the new system, thus improving the accuracy and usefulness of this entire data-set. Over 17000 of these assertions involve some specification of the stage of lethality and of these around 13000 proved amenable to scripted migration to the new system. This leaves around 4000 requiring manual review of free text phenotype descriptions before a sensible choice can be made about how to annotate using the new system.

## Discussion

Formal definitions in the DPO mostly follow patterns established for other phenotype ontologies [10-12]. In the DPO, we assert as little classification as possible and rely on a reasoner to infer classifications. These inferred classifications are instantiated in the '-simple’ release versions (see 1). Where classifications are asserted it is because the DPO or external referenced ontologies currently have insufficient formalisation to infer them. This approach is in contrast to most other phenotype ontologies which have kept their asserted classifications largely in-tact, although reasoning has been used to assess the validity of these assertions [25].

The biggest divergence between DPO and other phenotype ontologies is the system for specifying mortality rates during development. The Human Phenotype Ontology (HPO) [26], the Mammalian Phenotype Ontology (MPO) [27] and the (nematode) Worm Phenotype Ontology (WPO) [28] all include terms for recording the stage of death. Some of these terms have formal definitions, but these are not useful for reasoning about the amount or timing of death in populations. One example of increased clarity that adoption of our system in other phenotype ontologies would bring is that is that it would make explicit the different uses of the term 'lethal’ by different communities of biologists. For example, in contrast to the specialized meaning this term has to *Drosophila* biologists, 'lethal’ in the worm phenotype ontology covers increased mortality at any point in the life cycle including mature adulthood. It could therefore be formally defined following the pattern used for *'increased mortality’* in the DPO.

The only other ontological framework for recording mortality rates that we are currently aware of, an impressively detailed and well-axiomatised proposal from Sanatana and colleages [29], is not suited to our needs: it is based on a different upper ontology to the DPO; it assumes that death is due to injury or disease; and it does not include axioms for reasoning about relative timings.

The definition pattern we propose in this system is moderately complicated compared to the other formalisms we use. Adding new terms using these patterns manually is more laborious and potentially error-prone than for simpler formalisms. But the pattern is highly stereotyped and each definition only contains two elements that change: the age of animals the population referred to and the proportion of animals in that population that die (excluding the wild-type death rate). It would therefore be easy to specify a template-based system for creating new terms along the lines of the TermGenie system developed by the Gene Ontology (http://go.termgenie.org/).Another potential drawback is the scaling of reasoning time as the ontology grows. The formalisation uses elements, such as inverse object properties and universal quantification (*only*), that are outside the EL profile of OWL2 [30]. As a result, it is not possible to completely classify the DPO with fast, scalable, concurrent reasoners available for OWL2EL such as ELK [31]. However, at the current scale, classification is sufficiently fast, under 40 seconds with the DL reasoner HermiT (http://www.hermit-reasoner.com/), that it can be run frequently during ontology development.

### Future work

Efforts to increase the number of formal definitions are ongoing. We have deliberately taken a conservative approach to adding formal definitions specifying necessary and sufficient conditions for class membership. It is important to guard against applying simple definition patterns that make membership of a class overly broad, leading to serious errors in annotation grouping and query results. As a result, 23% of terms in the DPO still lack formal definitions specifying necessary and sufficient conditions for class membership.

Among these are phenotypic classes such as *touch sensitive* that group phenotypes according to performance in some assay but are agnostic about the underlying etiology. In other cases, the etiology is clear, but the phenotype is still hard to formalize. Classic segmentation phenotypes are a good example of this. A gap phenotype is defined as: 

“Embryonic/larval segmentation phenotype that is the complete loss of a contiguous stretch of 2 or more segments.”

We can record that a defect in the process of segmentation is a necessary condition for classification as a gap phenotype, but defining additional clauses for a complete set of necessary and sufficient conditions for class membership is much more challenging. We have also, so far, avoided the challenge of defining complex phenotypes that have multiple features, such as the *Minute* (FBcv_0000443) phenotype, which combines slow development and short bristles.

## Conclusions

The presence of textual definitions for all terms in the DPO ensures the accuracy of future curation with this ontology both by FlyBase and by any other group who use it. The process of composing both textual and formal definitions for DPO terms has involved extensive analysis of existing annotations. As a result of this, we have improved the DPO to more closely fit curator need, and improved the existing annotation set to be more consistent and coherent.

Composing formal definitions for terms in the DPO using high-quality, external ontologies, such as the GO, has allowed us to leverage classification and other formalisations in these ontologies to classify phenotypes. As a result, 85% (258/305) classifications are inferred rather than asserted. This has resulted in much more accurate and complete grouping of phenotype annotations using the DPO. For example, using the old manual classification, a query of the current FlyBase CHADO database [32] for *stress response defective* (FBcv_0000408) phenotypes finds only 344 phenotypes (481 alleles), whereas with the latest DPO release it finds 859 phenotypes (910 alleles).

The formalisation presented here increases the possibilities for sophisticated and accurate queries to be made against the very large, rich dataset of DPO annotations curated and maintained by FlyBase. One way to do this is to pipe the results of OWL queries of the DPO into SQL queries of the open FlyBase CHADO SQL server maintained by FlyBase. A guide for how to access DPO annotated phenotypes in the FlyBase CHADO database, including sample SQL queries, can be found at https://sourceforge.net/p/fbcv/wiki/chado_query_guide/. A similar approach has recently been successfully used to predict gene function from phenotype annotation in FlyBase and other model organism databases [33]. Similar formalisations for other phenotype ontologies have been successfully used for cross-species prediction of gene function and to search for disease models [10,16,34].

So far, the DPO has only been used by FlyBase, but it is freely available under an open source license, and there is no reason that it could not be used more widely and extended to cover a broader range of phenotypes in collaboration with interested parties.

## Methods

The DPO was largely developed in OBO format as part of a range of controlled vocabularies sharing the FBcv ID namespace. It still shares this ID namespace but is now available as a separate file. Most development has used OBOEdit, but with continuous conversion to OWL during development so that the results can be checked, browsed and queried with an OWL reasoner. Conversion to OWL and import of modules from from other ontologies was done using the OBO Ontology release Tool (OORT) (https://code.google.com/p/owltools/wiki/OortIntro) under the control of a continuous integration (CI) server which was also used to trigger a suite of Perl syntax checking and derivation scripts. As a result, each commit to our ontology development repository triggered syntax and consistency checking, rolled new textual definitions and generated various flavours of DPO file in OBO and OWL. The recent introduction of data-properties and universal quantification to our formalisation has prompted a shift to develop some components of the DPO in OWL - with the different component files being knitted together by OORT during CI. We anticipate that development will move fully to OWL in the near future.

Our module generation strategy works as follows: For every term from an external ontology used in a DPO axiom we import all terms and axioms on paths to root from a pre-reasoned version of the external ontology using OORT.

See Table 1 for URLs for accessing DPO files, terms and information.

*Conventions used in this paper*: All OWL entities in are identified in free text using their label in italics (for classes) or bold (for Object Properties), followed by their OWL short form ID in brackets. Following the OBO foundry ID standard (http://www.obofoundry.org/id-policy.shtml) a full URI can be generated by prepending http://purl.obolibrary.org/obo/ to his ID. In most cases this URI will resolve to OntoBee (http://www.ontobee.org/), returning XML if accessed programatically. OBO IDs can be derived by converting the underscore in a short-form ID to a colon. All formal axioms are expressed in OWL Manchester syntax (OWL-MS). OWL-MS keywords are italicised. Object properties (relations) are in bold. The names of OWL entities (e.g. classes, object properties) are quoted only if they contain spaces.

*Allen interval algebra*: For reasoning about relative timing, we use relations based on a subset of the Allen interval relations: precedes (p), 'preceded by’ (P), met (m), 'met by’ (M), during (d) starts (e), finishes (f). p *inverseOf* P, m *inverseOf* M. Transitive properties: M, P, d. Key axioms from the Allen composition table are represented using OWL property chains.

## Endnotes

^a^ 'has extra parts of type’ is a PATO relational quality. We use this relational property in combination with the relation **towards**. It would be simpler to use a single relation in place of this combination, but we use this pattern order to conform to standards established for other phenotype ontologies.

^b^ Age is a non-rigid property - individuals retain their identity when it changes. Using such properites has some modelling disadvantages [35]. For example, it is not possible in OWL to both track the identity of individuals as they age, and to classify them appropriately under classes whose formal definition includes an age restriction.

^c^ The range of **has_increased_mortality_rate** is a whole number (datatype short) between 0 and 100 inclusive. It would make more sense to allow fractional percentage values, but current limitations of reasoners exclude this option.

## Competing interests

The authors declare that they have no competing interests.

## Authors’ contributions

GVG proposed the initial set of formalisations, except those for recording the timing of lethality. These were then refined by DOS in consultation with FlyBase genetic curators (SJM, GM, PAM, LP and RS) and NB. Formal definitions of terms for recording the timing of lethality were designed by DOS in consultation with FlyBase genetic curators. SJM, GM, PAM, LP, RS and NB all made significant contributions to the textual definitions of many terms and to validating the structure of the ontology. Their work also ensured that the new definitions complied with current and past usage. KF played a critical role in retrofitting phenotype annotations in FlyBase following changes to the DPO. This paper was primarily written by DOS with edits and suggestions from all other authors. All authors read and approved the final manuscript.
